# Growth-Inhibitory Activity of a Clerodane Diterpene from *Callicarpa americana* Leaf Extracts and Its Derivatives against *Cutibacterium acnes*

**DOI:** 10.1021/acsinfecdis.5c01116

**Published:** 2026-04-27

**Authors:** Ben Kittleson, Sunmin Woo, Lewis Marquez, Michael J. Zeiler, Isabel Daher, Christian Melander, Cassandra L. Quave

**Affiliations:** 1 Department of Biology, 1371 Emory University, 1510 Clifton Rd. NE, Room 2006, Atlanta, Georgia 30322, United States; 2 Center for the Study of Human Health, 1371 Emory University, 1557 Dickey Drive, Anthropology 306, Atlanta, Georgia 30322, United States; 3 Molecular and Systems Pharmacology Program, 1371 Emory University, 615 Michael St., Whitehead 115, Atlanta, Georgia 30322, United States; 4 Jones Center at Ichauway, Newton, Georgia 39870, United States; 5 Department of Chemistry and Biochemistry, 6111 University of Notre Dame, Notre Dame, Indiana 46556, United States; 6 Microbiology and Molecular Genetics Graduate Program, 1371 Emory University, 1510 Clifton Road NE, Atlanta, Georgia 30322, United States; 7 Department of Dermatology, 1371 Emory University, 615 Michael St., Whitehead 105L, Atlanta, Georgia 30322, United States; 8 Herbarium, 1371 Emory University 1462 Clifton Road, Atlanta, Georgia 30322, United States

**Keywords:** *Cutibacterium acnes*, acne, clerodane diterpene, *Callicarpa americana*, antimicrobial agents, growth inhibition assay

## Abstract

*Cutibacterium acnes* is a Gram-positive bacterium implicated in acne vulgaris, a common inflammatory skin disease. Rising antibiotic resistance in *C. acnes* highlights the need for new therapeutic agents. *Callicarpa americana* L., a shrub native to the southeastern United States, has a history of use in Native American traditional medicine. Bioassay-guided fractionation of *C. americana* leaf extracts led to the isolation of 12­(S),16ξ-dihydroxycleroda-3,13-dien-15,16-olide (compound **1**), a clerodane diterpene. Compound **1** exhibited growth-inhibitory activity against *C. acnes* (MIC: 8 μg/mL). A route to full synthesis of compound **1** and its derivatives was previously achieved by our groups, and we demonstrate that the growth inhibitory activities of synthetic compound **1** is equivalent to that of **1** isolated from *C. americana*. Furthermore, analogs of **1** were evaluated against *C. acnes*, with two structurally similar compounds demonstrating slightly reduced efficacy, supporting previous findings that the C-12 substituent, but not its stereochemistry, is important to the compound’s activity. These findings expand knowledge of the antimicrobial properties of compound **1**, provide the first report of any clerodane diterpene compound with activity against *C. acnes*, and serve as an important example of the role of how plant natural products can aid the drug discovery pipeline.

## Graphical abstract

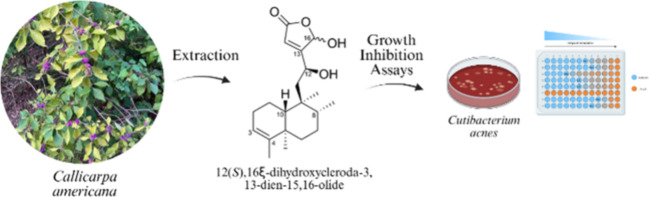

## Introduction

Acne vulgaris is a widespread inflammatory disease that affects the pilosebaceous units of the skin on the face, neck, and torso.^1,2^ A systematic analysis of acne vulgaris rates across the globe over two decades estimated that it is the eighth most common skin disease globally, affecting ∼9.4% of the global population at any given time.^3^ The disease typically presents with lesions—which can often be painful—and can vary greatly in severity.^1^ While in the vast majority of cases, acne vulgaris does not pose a serious threat to a patient’s health, severe disease is often associated with strong negative social impacts, including decreased self-confidence, mental distress related to body image and sexuality, and difficulties managing emotions and daily activities.^4^ Furthermore, acne is the only skin disease whose outcome has been implicated as a risk factor for suicide, especially in men.^5^ Therefore, management of acne vulgaris is an important step in preventing negative mental health outcomes.

A key factor in the pathology and progression of acne vulgaris is the normal skin flora *Cutibacterium acnes* (formerly *Propionibacterium acnes*). *C. acnes* is a Gram-positive, anaerobic bacterium that colonizes the pilosebaceous units of human skin.^6^ While *C. acnes* is typically associated with the regulation of skin health, alterations in *C. acnes* prevalence compared to standard levels, such as the increase that typically occurs during puberty, is a key factor in the proliferation of acne vulgaris, and can cause the bacterium to become an opportunistic pathogen.^6–8^ Lipases secreted from *C. acnes* are known to hydrolyze free fatty acids on the skin, which become proinflammatory and acnegenic in the pilosebaceous follicles.^9^ In addition, two major components of *C. acnes* cell walls have been demonstrated to activate toll-like-receptors in patients with acne vulgaris, which in turn activates multiple pathways that produce various proinflammatory cytokines, such as tumor necrosis factor-α, interleukin-1β, interleukin-6, and interleukin-8.^10,11^ In addition to its major role in acne vulgaris, *C. acnes* also presents issues in various infection types, particularly in postsurgical infections.^12^ These infections are particularly dangerous because of their delayed appearance of symptoms, inability to be rapidly cultured, and high morbidity. Current frontline therapies for acne vulgaris that specifically target *C. acnes* include benzoyl peroxide, topical retinoids, and various antibiotics, particularly tetracycline antibiotics.^13–17^

Previous work has demonstrated that multiple fractions from leaf extracts of *Callicarpa americana* exhibited strong inhibition against *C. acnes*, warranting further investigation of *C. americana* and its active therapeutic agents.^18^ *Callicarpa americana*, commonly known as the American beautyberry, is a shrub of the Lamiaceae family native to the Southeastern United States. *C. americana* has a long history of use in Native American traditional medicine systems—including those of the Seminole, Alabama, Choctaw, Koasati, and Creek tribes—with uses that ranged from treating fevers to skin cancer, among others.^19,20^

12­(*S*),16ξ-dihydroxycleroda-3,13-dien-15,16-olide (compound **1,** Figure 1) is a clerodane diterpene compound first isolated from the leaves of *C. americana* in 2007 by Jones et al.^21^ Our 2020 study of *C. americana* leaf extracts demonstrated that **1** was able to resensitize methicillin-resistant *Staphylococcus aureus* (MRSA) to β-lactam antibiotics.^22^ Compound **1** inhibited MRSA growth at concentrations of 4 μg/mL when combined with 2 μg/mL of oxacillin, while also demonstrating inhibitory activity alone at concentrations as low as 16 μg/mL, with an IC_50_ against HaCaT cells above these concentrations (64 μg/mL). Since **1** had a low reported yield from the *C. americana* crude leaf extracts (<0.1%), we developed a route to access **1** and analogs through total synthesis.^23^

**Figure 1:**
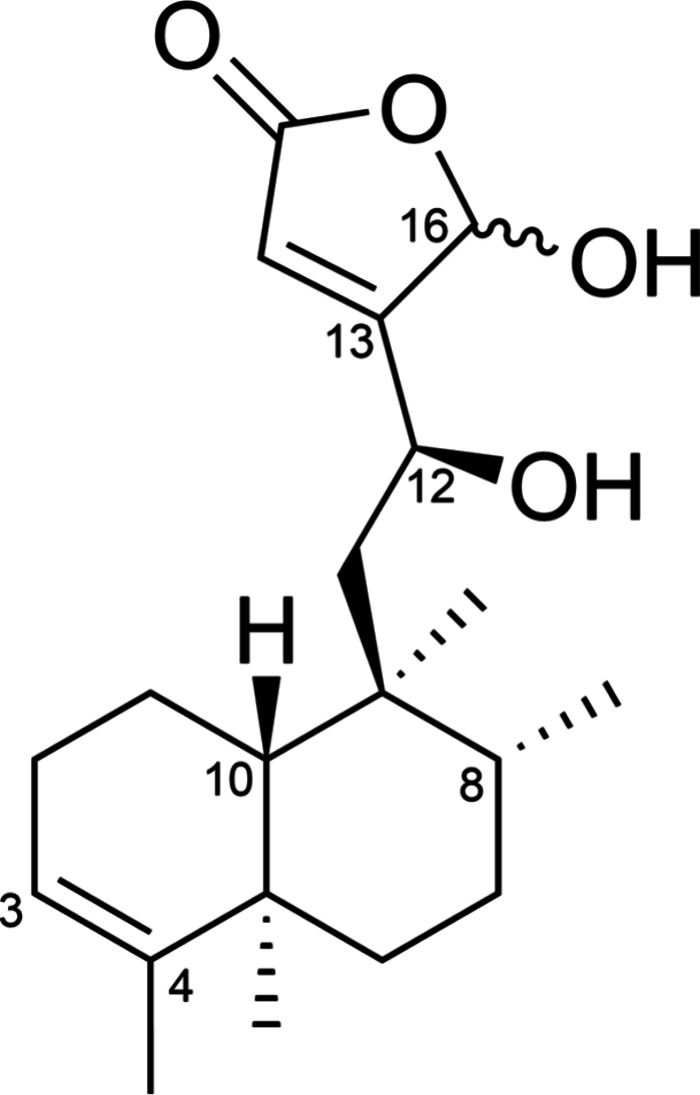
Chemical structure of 12­(*S*),16ξ-dihydroxycleroda-3,13-dien-15,16-olide, compound (1).^23^

Using a process of bioassay-guided fractionation, **1** was hypothesized to be a key component of the previously observed antiacne properties of *C. americana* leaf extracts (MIC: 8 μg/mL). With little existing literature on the antibacterial activity of **1** and other related compounds, a preliminary structure activity relationship analysis was conducted using related synthetic compounds from our previous report to identify potential important components of the compound’s structure to guide future analog synthesis.^23^ This analysis demonstrated the first report of antiacne activity in any clerodane diterpene compound, provided further support for the identification of **1,** and helped identify key components of **1**’s structure that may contribute to its antimicrobial activity.

## Results and Discussion

### Chemical Separation

Initial extraction of 520.7 g of *Callicarpa americana* leaves in 95% ethanol yielded 74.99 g of crude extract 2745 (14.40%) (Figure S1). Of this, 49.59 g of 2745 was subjected to liquid–liquid partitioning, yielding four partitions: hexane (2745B), ethyl acetate (2745C), *n*-butanol (2745D), and water (2745E). These partitions had yields of 5.19 g (10.46%), 5.41 g (10.91%), 9.42 g (19.00%), and 19.48 g (39.28%), respectively (B, C, D, and E). 2745C was chosen for further separation based on previous work with *C. americana* leaf extracts.^18^

A total of 4.99 g of fraction 2745C was separated using flash chromatography, yielding 93 total unique fractions (Figure S2). These fractions were subjected to a MIC assay screening, which was used to combine these fractions into only nine total fractions (2745C–F1 through 2745C–F9). Fraction 2745C–F2 yielded 237.9 mg (4.76%) and displayed a MIC against *C. acnes* of 16 μg/mL. Fraction 2745C–F4 yielded 641.6 mg (12.85%) and had similar growth-inhibitory activity against *C. acnes*. Given this, both fractions were chosen for further separation via High-Performance Liquid Chromatography (HPLC).

63.2 mg of fraction 2745C–F4 was then separated using reverse-phase HPLC to yield 40 fractions, which were then recombined based on the chromatogram to produce 16 total fractions (Figure S3). Fraction 2745C–F4–PF7 had the greatest yield (72.10%) and was chosen for further separation based on the growth inhibition data. Using a dual solvent-system gradient, a total of 35.0 mg of fraction 2745C–F4–PF7 was then separated via normal-phase HPLC, yielding seven total fractions (Figure S4). Fraction 2745C–F4–PF7-SF4 had the greatest yield (90.54%).

### Structural Identification

LC-MS data were obtained from a fraction generated from our previous isolation work with fraction 649C–F13–PF4-SF7, which was purported to contain the relatively pure compound **1** (Figure S5).^18,22^ The 649C–F13–PF4-SF7 spectrum showed at least three major peaks (Rt values: 5.004, 6.013, and 7.788) present in the sample. One of these peaks matched closely with the observed *m*/*z* value of compound **1** (333.2071, Rt: 6.013). After completion of normal-phase HPLC, LC-MS data were obtained for both 2745C–F4–PF7-SF2 and 2745C–F4–PF7-SF4. While 2745C–F4–PF7-SF2 now showed only one major ion (*m*/*z*: 367.2636), 2745C–F4–PF7-SF4 still contained two major ions (*m*/*z* values: 333.2080 and 367.2647, although far fewer trace ion peaks were reported in the sample compared to 649C–F13–PF4-SF7). The compound with an *m*/*z* value of 333.2080 was hypothesized to be the active component of 2745C–F4–PF7-SF4, given that it was present in the active subfraction, while that the other compound (*m*/*z*: 367.2636) was also present in the 2745C–F4–PF7-SF2 which had significantly reduced antimicrobial activity. An ^1^H NMR spectrum of 2745C–F4–PF7-SF4 was then collected. ^1^H NMR signals of compound **1** were detected and compared with the corrected literature values to validate this hypothesis.^22,23^ Based on the combination of ^1^H NMR and mass spectrometry data, **1** was identified as the major component of 2745C–F4–PF7-SF4.

### ^1^H NMR

2745C–F4–PF7-SF4 containing compound (**1**) (Figure S6). Amorphous solid. ^1^H NMR (600 MHz, CDCl_3_). Peaks of compound **1** were selectively chosen. δ 6.07 (1H, br s, H-16), 6.03 (1H, s, H-14), 5.17 (1H, s, H-3), 4.73 (1H, m, H-12), 2.07 (2H, br, H-2), 1.90 (1H, m, H-11a), 1.84 (1H, m, H-1a), 1.74 (1H, m, H-6a), 1.69 (1H, m. H-10), 1.56 (3H, s, H-18), 1.53 (1H, m, H-8), 1.51 (1H, m, H-11b), 1.47 (1H, m, H-1b), 1.45 (2H, m, H-7), 1.19 (1H, m, H-6b), 0.99 (3H, s, H-19), 0.78 (3H, d, *J* = 6.6 Hz, H-17), 0.73 (3H, s, H-20).

### Growth Inhibition Assays

Testing of the initial fractions generated from the separation of partition 2745C via flash chromatography revealed two fractions (2745C–F2 and 2745C–F4) with similar inhibitory activity against *C. acnes* (MIC: 16 μg/mL). 2745C–F4 was chosen for the initial further exploration due to its higher yield. Further separation of 2745C–F4 via both normal-phase and reverse-phase HPLC, accompanied by subsequent growth inhibition assays identified fraction 2745C–F4–PF7-SF4 as having the lowest MIC value (8 μg/mL) against *C. acnes* (Figure 2). This value is similar to the reported MIC for the clerodane diterpene compound previously isolated from *C. americana* (**1**) that resensitized MRSA to *ß*-lactams.^22^ While new schemes of fractionation and isolation were conducted to yield **1**, these findings build on previous work with *C. americana* to demonstrate additional evidence of **1**’s antibacterial activity against Gram-positive bacteria.^22^

**Figure 2:**
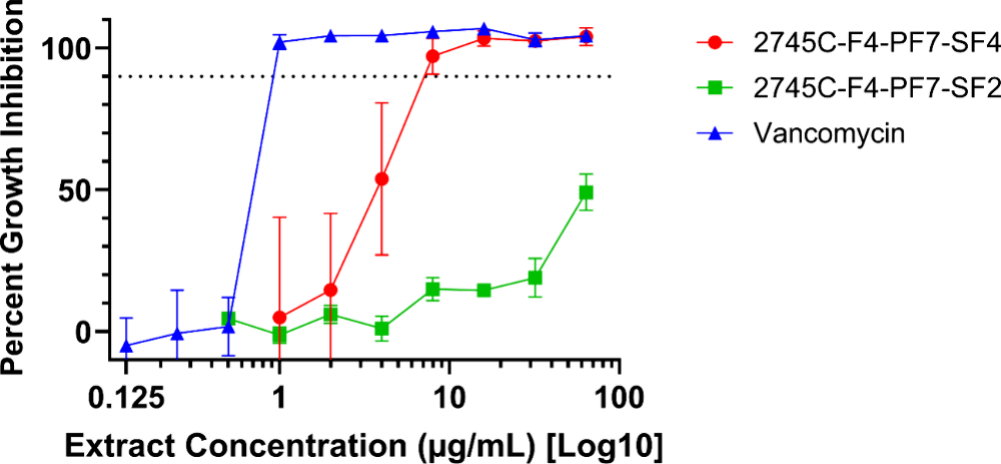
Growth-inhibitory activity of *Callicarpa americana* leaf fractions. Percent growth inhibition data relative to a DMSO vehicle control is shown for fractions 2745C–F4–PF7-SF4 and 2745C–F4–PF7-SF2 (acquired from *C. americana* leaf extracts) against *C. acnes* strain ATCC-6919. Vancomycin was used as a positive antibiotic control. All concentrations were conducted in triplicate with at least one additional biological replicate plated on separate days. Error bars represent standard deviations. The *x*-axis is log transformed to better visualize trends in inhibitory activity. The dotted horizontal line represents 90% inhibition.

Four additional *C. acnes* clinical isolates were examined and displayed varying levels of susceptibility to 2745C–F4–PF7-SF4 (Figure 3). Strains B104.6 and B104.7 showed identical MIC values (8 μg/mL) when compared to ATCC-6919 (8 μg/mL), while B104.5 showed a marginal reduction in growth inhibition (MIC: 16 μg/mL). Growth inhibition of B104.2 was marginal if present at all for all concentrations tested. A key difference between these isolates was ribotype: B104.2 had a different ribotype (RT3) than all the other strains, including ATCC-6919, all of which were RT1.^24^ Future testing with more clinical isolates of various ribotypes could reveal important determinants for compound efficacy related to ribotype.

**Figure 3:**
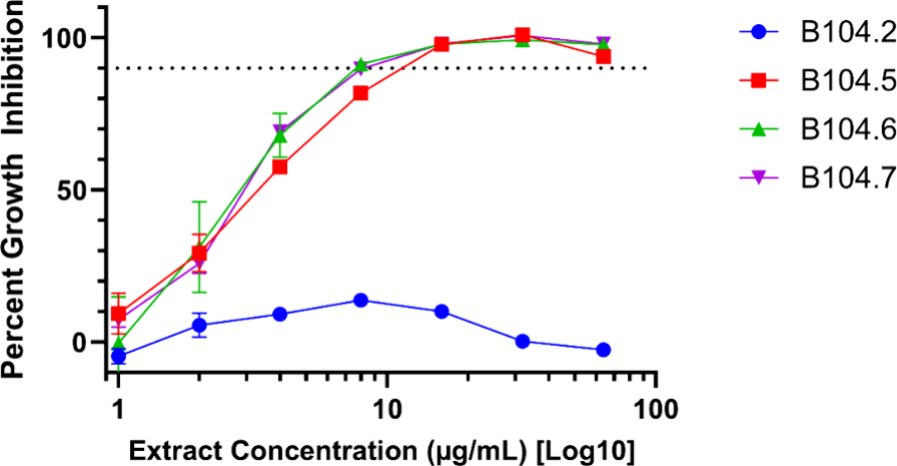
Growth-inhibitory activity of *Callicarpa americana* leaf fraction 2745C–F4–PF7-SF4. Percent growth inhibition data relative to a DMSO vehicle control is shown for fraction 2745C–F4–PF7-SF4 against multiple *C. acnes* clinical isolates. All concentrations were conducted in triplicate with at least one additional biological replicate plated on separate days. Error bars represent standard deviations. *x*-axis is log transformed to better visualize trends in inhibitory activity. The dotted horizontal line represents 90% inhibition.

### Structure–Activity Relationship Analysis

Growth inhibition assays of the synthetic epimeric mixture corresponding to compound **1** (**syn-1**), and three of its structural analogs from Zeiler et al. (**3**–**5**) (Figure 4 and Table S1), exhibited varying degrees of activity against *C. acnes*.^23^ As previously reported, both the isolated natural product (**1**) and the synthetic product (**syn-1**) exist as mixtures of epimers at C-16, as the absolute configuration at this position remains unsolved.^23^ Consistent with our prior work on MRSA, **syn-1** displayed similar inhibitory activity at all tested concentrations (1 μg/mL–64 μg/mL) when compared to 2745C–F4- PF7-SF4, with an equivalent MIC value (8 μg/mL). Compound **2**, a diastereomer of **1**, had a 2-fold increase in MIC value (16 μg/mL) compared to **1**. Compound **3** displayed a 4-fold increase in its MIC compared to **syn-1**, while **4** demonstrated no significant inhibitory activity, even at the highest concentration tested (64 μg/mL) (Table 1).

**Figure 4:**
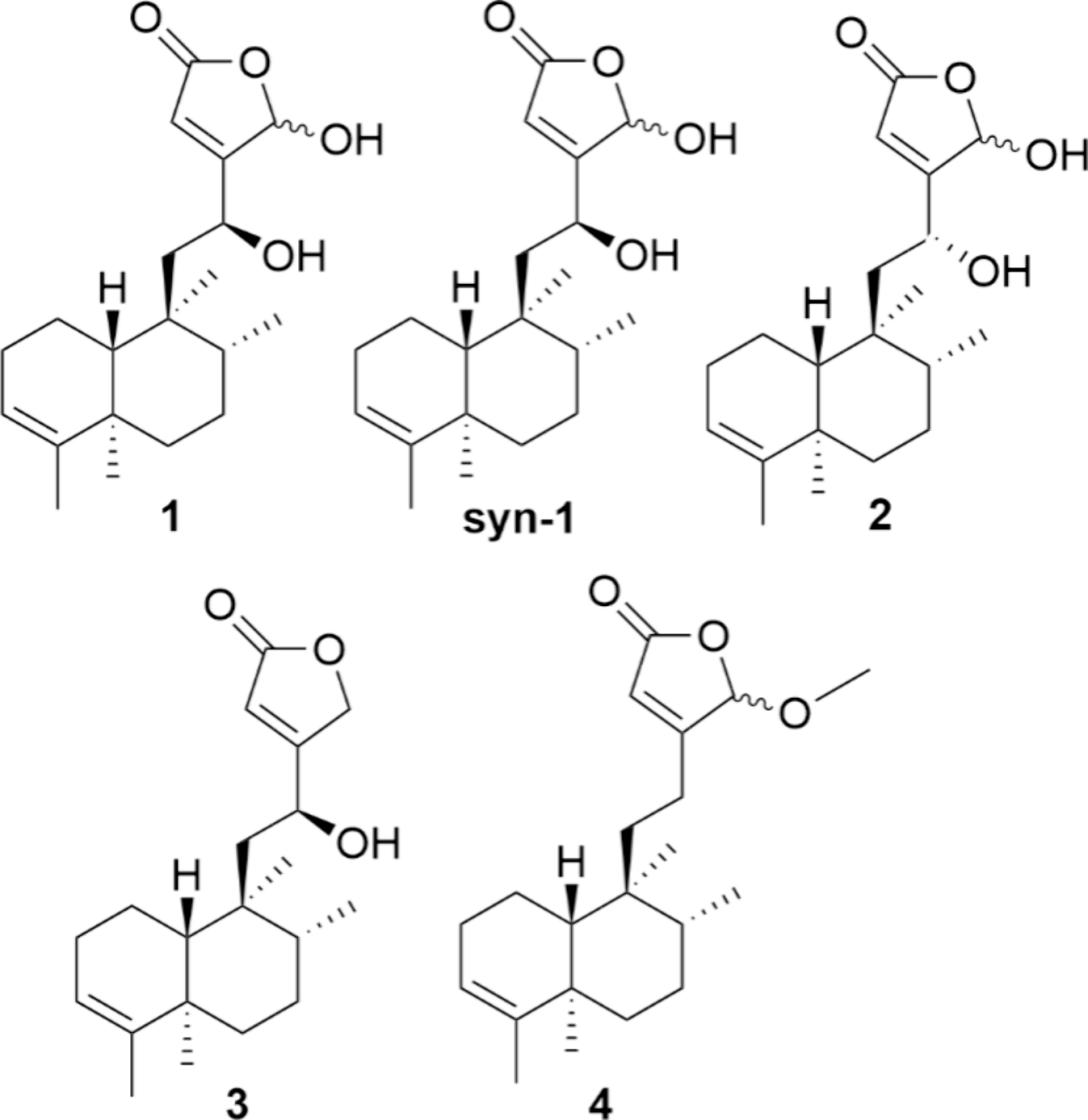
Chemical structure of the isolated natural product­(**1**)­and synthesized compounds (**syn-1, 2–4**). All five compounds shown were tested for their growth-inhibitory activity against *C. acnes*. Structure of **1**, its synthesized compound (**syn-1**) and three synthetic analogs (**2–4**) are also shown. It should be noted that **1** (isolated natural product) and **syn-1** (synthetic product) represent equivalent epimeric mixtures at C-16, as the absolute configuration at this position remains unresolved. The synthetic material was previously shown to match the natural product by spectroscopic analysis.^23^

**Table 1:** Minimum Inhibitory Concentrations (MIC) of Bioactive Synthesized Compounds^a^

Compound	MIC (μg/mL)
1, 2745C–F4–PF7-SF4	8
syn-1	8
2	16
3	64
4	>64

a Minimum inhibitory concentrations (MIC) values shown for 2745C–F4–PF7-SF4 (**1**) and synthesized compounds against *C. acnes*. All concentrations were conducted in triplicate with at least one additional biological replicate plated on separate days.

The structure–activity relationship (SAR) analysis provided some key insights into the importance of specific structures of compound **1** to its antimicrobial activity. While previous research established that stereochemical inversion of the OH of C-12 to yield compound **2** modestly increased standalone antimicrobial activity against MRSA,^23^ in this study we noted the opposite phenomenon and **2** displayed a slight decrease in growth inhibition of *C. acnes*, providing further evidence that this stereochemistry at C-12 has a minor effect on the compound’s inhibitory activity. Additionally, the ≥ 8-fold increase in MIC of compounds **3** and **4** with alterations to OH (either complete deoxygenation or masking as OMe), indicates that this section of the molecule is crucial for activity and future analogs efforts should focus on either bioisosteres of the butenolide or modification of the decalin core. This mirrors the results of previous work with compound **1**, which focused on its ability to resensitize MRSA to β-lactam antibiotics, specifically that the butenolide was critical for activity.^23^ Furthermore, this previous study reported minimal effect on the compound’s adjuvant activity at the C-12 carbon when the hydroxyl’s stereochemistry was inverted or the C–OH was reduced to C–H, but loss of activity was observed with modification of the C-16 carbon. Based on these findings, further work into this compound should probe for a potential conserved antibacterial mechanism against a wider array of Gram-positive bacteria.

## Conclusion

The extraction and bioassay-guided fractionation of *Callicarpa americana* leaf extracts led to the successful identification of 12­(*S*),16ξ-dihydroxycleroda-3,13-dien-15,16-olide (compound **1**) as an inhibitor of *Cutibacterium acnes* growth. Compound **1** is a clerodane diterpene previously isolated from the leaves of *Callicarpa americana* with reported antibacterial and cytotoxic effects in MRSA and lung cells lines, respectively (MIC: 16 μg/mL, ED_50_: 2.4 μg/mL).^21, 22^ This study further supports the antibacterial activity of compound **1** by being the first to report growth-inhibitory activity of compound **1** against not only *Cutibacterium acnes* but any anaerobic or non-*Staphylococcus* bacteria.^21,22^ Additionally, this is the first report of any clerodane diterpene compound with any antimicrobial activity against *C. acnes*. This growth-inhibitory activity was demonstrated by not only the fraction (2745C–F4–PF7-SF4) containing compound **1** but also by the corresponding identical compound achieve through total synthesis (**syn-1**). While most of the clinical isolates tested showed similar levels of growth inhibition as the strain ATCC-6919 when exposed to 2745C–F4–PF7-SF4, isolate B104.2 showed little to no growth inhibition, which invites further investigation into the importance of specific phenotypes that are associated with inhibition of *C. acnes* by **1**, potentially including ribotype. Therefore, these findings support the need for further research into compound **1**’s therapeutic potential for acne vulgaris, and identify clerodane diterpenes as a class of compounds requiring further investigation for applications against acne.

Further *in vivo* testing of compound **1**, both alone and in combination with other therapies like retinoids or benzoyl peroxide, could provide crucial data about the practicality of compound **1**’s applications as a therapeutic agent. Additionally, mechanistic studies of compound **1**’s growth-inhibitory activity are also key to understanding its therapeutic potential. Given the previous work demonstrating inhibition of MRSA by compound **1**, elucidation of an antibacterial mechanism may support additional applications of compound **1** beyond acne vulgaris.

While compound **1**’s growth-inhibitory effects are likely a key component of its parent fraction’s similar properties, two other bioactive fractions (2745C–F2–PF7 and 2745C–F2–PF8) displayed high levels of growth inhibition against *C. acnes* but were not investigated further due to possessing a lower yield than 2745C–F4–PF7. These fractions require further investigation to fully characterize the antibacterial metabolome of *Callicarpa americana* leaf extracts.

In the face of growing antibiotic resistance and declining discovery of novel antibiotics, it is becoming increasingly important to discover and investigate new therapeutics for bacteria-mediated diseases like acne vulgaris. The findings in this study demonstrate how investigations of global traditional medicine systems and ethnobotany can help facilitate the discovery of natural products with therapeutic applications that potentially aid the drug discovery pipeline. While further research is needed to probe the efficacy and safety of compound **1**, the identification of compounds and previously understudied classes of compounds with relevant biological activities like this one is an important first step in the development of novel therapeutics for diseases like acne vulgaris.

## Methods

### Plant Material Collection and Extraction

*Callicarpa americana* leaves were collected from wild plants at various locations across Georgia and Florida and their identities were verified by botanist Dr. Tharanga Samarakoon of the Emory University Herbarium (Table S2). These samples were then processed to remove all nonleaf portions of the plant and dried in a dehumidified chamber. A Wiley Mill (Thomas Scientific, NJ, USA) was then used to grind these leaves into a fine powder using a 2 mm filtering mesh. Leaf material and powdered samples were both deposited as voucher samples in the Emory University Herbarium. Collection information and voucher accession numbers are detailed in the Supporting Information file (Table S2).

### Crude Extraction

The powdered *C. americana* leaf material was subjected to a double maceration for 72 h after suspension in 95% ethanol at a ratio of 1:10 (w/v). All material from the first and second macerations was vacuum filtered using a Buchner funnel and VWR Qualitative filter paper (VWR, PA, USA) grade 415 and 413 (coarse and fine, respectively) to remove the retentate. The filtrates from the first and second macerations were then combined, dried via a rotary evaporator, redissolved in distilled-H_2_O (diH_2_O), shell frozen using a bath of dry ice and acetone, and lyophilized overnight. After lyophilization, each extract was stored in scintillation vials at −20 °C. Material collected at different times and from different locations was identified by different extract numbers—extract 649 for leaves collected in 2017, extract 2745 for leaves collected in 2022, and extract 2771 for leaves collected in 2023, in case of any significant phytochemical variation between different populations. The overwhelming majority of the experimental work in this study was conducted using extract 2745 due to the abundance of material available.

### Partitioning

Crude extract 2745 was redissolved in diH_2_O, at a ratio of 33 g/L, and partitioned against equal volumes of hexanes, ethyl acetate, and water saturated *n*-butanol. Each partition was then dried via a rotary evaporator, redissolved in diH_2_O, shell frozen using a bath of dry ice and acetone, and lyophilized overnight. After lyophilization, each partition was stored in scintillation vials at −20 °C. Based on previous work with this extract, the ethyl acetate partition (2745C) was chosen for further separation.

### Flash Chromatography

Fraction 2745C was combined with Celite in a ratio of 1:4 (w/w) and dissolved in methanol (MeOH). This mixture was then dried via rotary evaporation before packing into a Teledyne solid-phase load cartridge. The cartridge was then loaded onto a CombiFlash Rf+ Lumen (Teledyne ISCO, NE, USA) flash chromatography system with a 24 g RediSep Rf Gold Silica column. A stepwise gradient mixture of three mobile phases (hexanes, ethyl acetate, and methanol) with a constant flow rate of 35 mL/min was used based on a previously established method to further separate fraction 2745C (Table S3).^18^ A total of 93 test tubes were obtained, which were then combined into nine total fractions, dried down using a rotary evaporator, and stored at −20 °C in scintillation vials (Figure S2).

### Reverse-Phase High-Performance Liquid Chromatography (HPLC)

Reverse-phase HPLC was used to further separate the fractions 2745C–F2 and 2745C–F4 obtained from flash chromatography. An Agilent Technologies 1260 Infinity II Preparative LC System (Agilent, CA, USA) was used to carry out this separation. A dual solvent system with a stepwise gradient mixture was employed with 0.1% formic acid in H_2_O (v/v) (A) and 0.1% formic acid in acetonitrile (ACN) (v/v) with an Agilent Eclipse XDB-C18 (30 × 250 mm) column and a flow rate of 42.5 mL/min (Table S4). An Agilent Technologies 1200 Infinity Series Diode Array Detector was used to detect absorbance at 214 and 254 nm. Sample material was dissolved in approximately 20 mg/mL of MeOH and filtered using an Agilent Technologies Polytetrafluoroethylene Echonofilter (0.2 μm) before injection. Fractions of these two HPLC runs were collected in 30-s time-slices and then recombined to create nine total fractions from 2745C–F2 and 16 total fractions from 2745C–F4 (Figures S3 and S7). These fractions were then dried down using a rotary evaporator and stored at −20 °C in scintillation vials.

### Normal-Phase HPLC

Normal-phase HPLC was used to further separate fraction 2745C–F4–PF7 obtained from reverse-phase HPLC. An Agilent Technologies 1260 Infinity II Analytical LC System (Agilent, CA, USA) was used to carry out this separation. A dual solvent system with a stepwise gradient mixture was employed with CH_2_Cl_2_(DCM):MeOH (97:3 v/v) and isopropyl alcohol (100%) with a YMC-Pack SIL-06 (10 × 250 mm) column heated to 30.0 °C and a flow rate of 4.00 mL/min (Table S5). UV detection was performed at four wavelengths: 254, 280, 320, and 365 nm. Sample material of 2745C–F4–PF7 was dissolved in a mixture of DCM and MeOH (95:5 v/v) at a concentration of 50 mg/mL and filtered using an Agilent Technologies Polytetrafluoroethylene Echonofilter (0.2 μm) before injection. Fractions of these HPLC runs were collected based on diode array detector, and seven total fractions were created (Figure S4). These fractions were then dried down using a rotary evaporator and stored at −20 °C in scintillation vials.

### ^1^H NMR

All samples for NMR analysis were prepared in deuterated chloroform (CDCl_3_), with solvent chemical shifts reported in ppm using established standards.^25^ ^1^H NMR spectra were obtained using a 14.1T Varian-INOVA 600 MHz instrument. All spectra were acquired at a spectral width of −2–14 ppm with 16 total scans, a relaxation delay of one second, and a pulse angle of 45 deg. Further visualization and analysis of NMR spectra were completed using the software MestReNova 14.0 (Mestrelab Research, Santiago de Compostela, Spain).

### LC-MS Analysis

All samples were dissolved in HPLC grade MeOH at concentrations of 0.02 mg/mL and filtered using a Regenerated Cellulose (RC) Syringe Filters (0.2 μm). An Agilent 1290 Infinity II UHPLC system coupled to an Agilent 6545XT QTOFMS and a Dual AJS ESI Ion Source (Agilent Technologies). Liquid chromatographic separations were performed on a Zorbax Eclipse XDB-C18 (100 × 2.1 mm, 1.8 μm) column at 40 °C. The sample organizer was held at 15 °C. The mobile phase consisted of 0.1% formic acid in H_2_O (A) and 0.1% formic acid in ACN (B). A stepwise gradient of the following conditions was used: 5–5% B (0.0–0.5 min); 5–90% B (0.5–6.0 min); 90–100% B (6.0–9.0 min); 100–100% B (9.0–10.5 min); 100–5% B (10.5–10.6 min); and 5–5% B (10.6–12.0 min). Using 2.0 μL injections, samples were analyzed in the negative ion mode. The electron spray ionization conditions used a capillary voltage of 4.0 kV, a nozzle voltage of 2000 V for negative mode, a fragmentor voltage of 100 V, a drying gas (nitrogen) temperature of 325 °C with a flow rate of 13 L/min, and a sheath gas (nitrogen) temperature of 275 °C with a flow rate of 12 L/min. Nebulizer pressure was held at 35 °C. An MS range of *m*/*z* 100–1700 and an MS2 range of *m*/*z* 50–1700 were employed in Auto-MS/MS mode, at 7 spectra/s and 5 spectra/s, respectively, with an isolation with of ∼ 1.3 *m*/*z*. The collision energy was determined based on the precursor charge and *m*/*z* values. The precursor ions were set to a maximum of five per cycle and an absolute threshold of 500, with active exclusion after 3 scans. MassHunter Workstation Acquisition B.10.00 software and MassHunter Qualitative Analysis 10.0 software (Agilent Technologies) were used for data visualization and analysis.

### Bacterial Culture

*C. acnes* growth was conducted using CLSI M11 standards for anaerobic bacteria.^26^ *C. acnes* strains were obtained from American Type Culture Collection (strain ATCC-6919) and clinical samples from UCLA courtesy of Dr. Laura Marinelli and cultured for either 72 h (ATCC-6919) or 96 h (clinical isolates) at 37 °C on tryptic soy agar (TSA) plates with 5% sheep’s blood in an EZ Anaerobe Chamber with GasPak EZ Anaerobe container sachets. An anaerobic indicator was placed in the chamber and observed for a color change from pink to white to confirm an anaerobic environment. After 72 h/96 h, plates were analyzed for colony morphology to confirm plates were a pure culture of *C. acnes*, and single colonies were used to inoculate 6–7 mL of Brain Heart Infusion broth with 1% dextrose (BHId) in 14 mL snap cap tubes. These tubes were then incubated at 37 °C for 72 h/96 h in the same anaerobic growth conditions.

### Growth Inhibition Testing & Structural Activity Relationship Analysis

Specific CLSI guidelines do not exist for antimicrobial testing of *C. acnes*, so minimum inhibitory concentrations (MICs) were determined using a previously described method.^27^ Briefly, bacterial cultures in BHId were standardized after incubation using a BioTek Cytation-3 multimode plate reader and additional BHId broth to 5 × 10^7^ CFU/mL (0.05 optical density at 590 nm) based on previous work with *C. acnes.* ^18^ To determine the MIC of various fractions of the *C. americana* leaf extracts, assays were conducted in sterile 96-well plates (Falcon 35–3705). Fractions dissolved in 4 mg/mL stock solutions of dimethyl sulfoxide (DMSO) were combined with BHId and bacteria at 5 × 10^7^ CFU/mL in BHId for a final well volume of 200 μL. Serial dilutions were performed to create final concentrations of each fraction ranging from 64 μg/mL to 0.5 μg/mL. Vehicle controls (DMSO), antibiotic controls (Vancomycin -4 μg/mL), and untreated controls were included with each assay. DMSO was <2% (v/v) in all wells tested. All treatment concentrations were tested in triplicate. Plate readings were taken at 600 nm using the BioTek Cytation-3 multimode plate reader at 0 h and after either 72 h (ATCC-6919) or 96 h (clinical isolates) in the previously described anaerobic conditions at 37 °C. The MIC was determined by examining the plate for the lowest concentration of the compound or extract being tested in which no visible bacterial growth was present, equivalent to a 90% inhibition of optical density as detected by plate reader. Percent growth inhibition calculations were conducted using changes in optical density from 0 to 72 or 96 h relative to the same value for the vehicle control to account for the color of extracts and reported as averages for the three replicates.^28^

Stock solutions of synthetic compounds (10 μM in DMSO, > 95% purity) were supplied by Dr. Christian Melander of Notre Dame University.^23^ For the structure–activity relationship analysis, the same growth inhibition assays were conducted, and compound **1** was tested at 64 to 1 μg/mL, while all other compounds (**syn-1, 2–4**) were tested at the same molar dilution factor, regardless of molecular weight. A total of five synthetic compounds were tested (Table S1).

### Statistical Analyses

All extracts were tested with three technical replicates, with at least two biological replicates on two separate days. Values are expressed as mean ± standard deviation. When applicable, data were analyzed using a two-way analysis of variance (ANOVA) with Tukey’s multiple comparison test using GraphPad Prism version 10.1.2 for Windows (GraphPad Software, San Diego, California, USA).

## Supplementary Material



## Notes

The Supporting Information file has been included here: The Supporting Information is available free of charge at https://pubs.acs.org/doi/10.1021/acsinfecdis.5c01116.

Structures of synthetic analogs of **1**, specimen collection information, flash chromatography and HPLC gradient elution methods, extract fractionation tree, flash chromatogram of 2745 partitions, HPLC chromatograms of 2745 fractions, LC-MS spectrum of **1**, ^1^H NMR spectrum of **1** (PDF)

## Abbreviations

BHId: Brain heart infusion broth with 1% dextrose
CFU: Colony forming unit
IC: Inhibitory concentration
MRSA: Methicillin-resistant *Staphylococcus aureus*
RT: Ribotype
TSA: Tryptic soy agar
