# Supplementary material for: Growth-Inhibitory Activity of a Clerodane Diterpene from Callicarpa americana Leaf Extracts and Its Derivatives against Cutibacterium acnes
Source: ACS Infect Dis. 2026 Apr 27;12(5):1662–8. doi: 10.1021/acsinfecdis.5c01116 (PMC13162267; doi:10.1021/acsinfecdis.5c01116)
Supplement: Supplementary file 1 [file id5c01116_si_001.pdf]

Supporting information

Growth-Inhibitory Activity of a Clerodane Diterpene from *Callicarpa americana* Leaf Extracts and its Derivatives Against *Cutibacterium acnes*

Ben Kittleson[1], Sunmin Woo[2], Lewis Marquez[3,4], Michael J. Zeiler[5], Isabel Daher[6], Christian Melander[5*], Cassandra L. Quave[2,7,8*]

[1]Emory University, Department of Biology, 1510 Clifton Rd. NE, Room 2006, Atlanta, GA, 30322, United States
[2]Emory University, Center for the Study of Human Health, 1557 Dickey Drive, Anthropology 306, Atlanta, GA 30322, United States
[3]Emory University, Molecular and Systems Pharmacology Program, 615 Michael St., Whitehead 115, Atlanta, GA 30322, United States
[4]Jones Center at Ichauway, Newton, Georgia 39870, United States
[5]University of Notre Dame, Department of Chemistry and Biochemistry, Notre Dame, IN 46556, United States
[6]Emory University, Microbiology and Molecular Genetics Graduate Program, 1510 Clifton Road NE, Atlanta, GA, 30322, United States
[7]Emory University, Department of Dermatology, 615 Michael St., Whitehead 105L, Atlanta, GA 30322, United States
[8]Emory University Herbarium, 1462 Clifton Road, Atlanta, GA 30322, United States

* Corresponding authors: cquave@emory.edu; cmelande@nd.edu

Table of contents

Table S1: Growth inhibition of *Cutibacterium acnes* by synthetic 12(*S*),16ξ-dihydroxycleroda-3,13-dien-15,16-olide and structural analogs S2
Table S2: *Callicarpa americana* leaf specimen collection information S3
Table S3: Flash chromatography gradient elution conditions S4
Table S4: HPLC solvent gradient method for 2745C-F2 and 2745C-F4 S5
Table S5: HPLC solvent gradient method for 2745C-F4-PF7 S5
Figure S1: Fractionation tree of extract 2745 S6
Figure S2: Flash chromatogram and separation scheme of 2745C S6
Figure S3: Reverse-phase HPLC chromatogram of 2745C-F4 S7
Figure S4: Normal-phase HPLC chromatogram of 2745C-F4-PF7 S7
Figure S5: Mass spectra of Callicarpa americana leaf fractions S8
Figure S6: ^{1}H NMR Spectrum of 2745C-F4-PF7-SF4 S9
Figure S7: Reverse-phase HPLC chromatogram of 2745C-F2 S10

Table S1: Growth inhibition of *Cutibacterium acnes* by synthetic 12(*S*),16ξ-dihydroxycleroda-3,13-dien-15,16-olide (syn-1) and structural analogs (2-4).

Compound ID Number	Structure	Molecular Weight (g/mol)	*C. acnes* MIC (µg/mL)
Compound **syn-1**	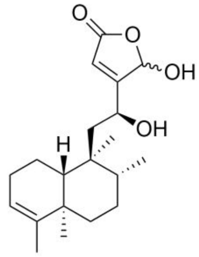	334.46	8
Compound **2**	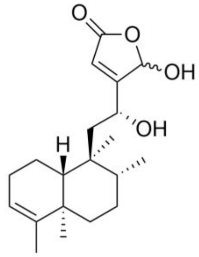	334.46	16
Compound **3**	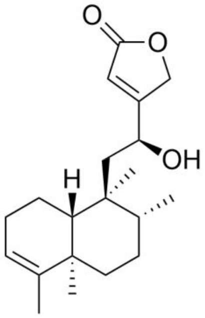	318.46	64
Compound **4**	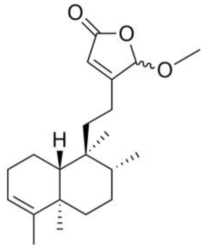	332.48	>64

Table S2: ***Callicarpa americana*** **leaf specimen collection information.**

Extract Number	GEO Accession Number(s)	Voucher Number(s)	Collection Date(s)	Location	Coordinates
649	22044, 22204, & 22205	TS-009 & TS-012	June-August 2017	DeKalb County, GA, USA	33.8281800000 N, −84.2872430000 W & 33.8036944440 N, −84.3230833330 W
2745	18293	RL-2022-2	October 2022	Tallahassee, FL, USA	30.55761373159117 N, -84.26169033997068 W
2771	17980	TS-101	July 2023	Newton, GA, USA	31.23937226609836 N, -84.46725437740263 W

Table S3: Flash chromatography gradient elution conditions.

Collection Volume (CV)	% Hexane	% Ethyl Acetate	% Methanol
0.0	100.0	0.0	0.0
2.0	100.0	0.0	0.0
1.7	99.0	1.0	0.0
1.7	98.0	2.0	0.0
1.7	96.0	4.0	0.0
1.7	91.9	8.1	0.0
2.1	85.1	14.9	0.0
6.6	68.1	31.9	0.0
7.0	35.9	64.1	0.0
7.0	0.0	100.0	0.0
8.3	0.0	100.0	0.0
0.1	0.0	100.0	0.0
1.7	0.0	99.0	1.0
1.7	0.0	98.0	2.0
1.7	0.0	96.0	4.0
1.7	0.0	92.0	8.0
1.7	0.0	84.0	16.0
1.7	0.0	70.0	30.0
8.3	0.0	70.0	30.0
1.7	0.0	0.0	100.0
6.0	0.0	0.0	100.0

Table S4: HPLC solvent gradient method for 2745C-F2 and 2745C-F4. A represents 0.1% formic acid in water, and B represents 0.1% formic acid in acetonitrile.

Time (minutes)	% A	% B
0.00	50	50
3.00	50	50
20.00	5	95
30.00	5	95
30.10	50	50
35.00	50	50

Table S5: HPLC solvent gradient method for 2745C-F4-PF7. % A represents % DCM/MeOH (97:3), while % B represents % isopropyl alcohol.

Time (minutes)	% A	% B
0.00	100	0
7.00	100	0
10.00	0	100
15.00	0	100
15.01	100	0
23.00	100	0

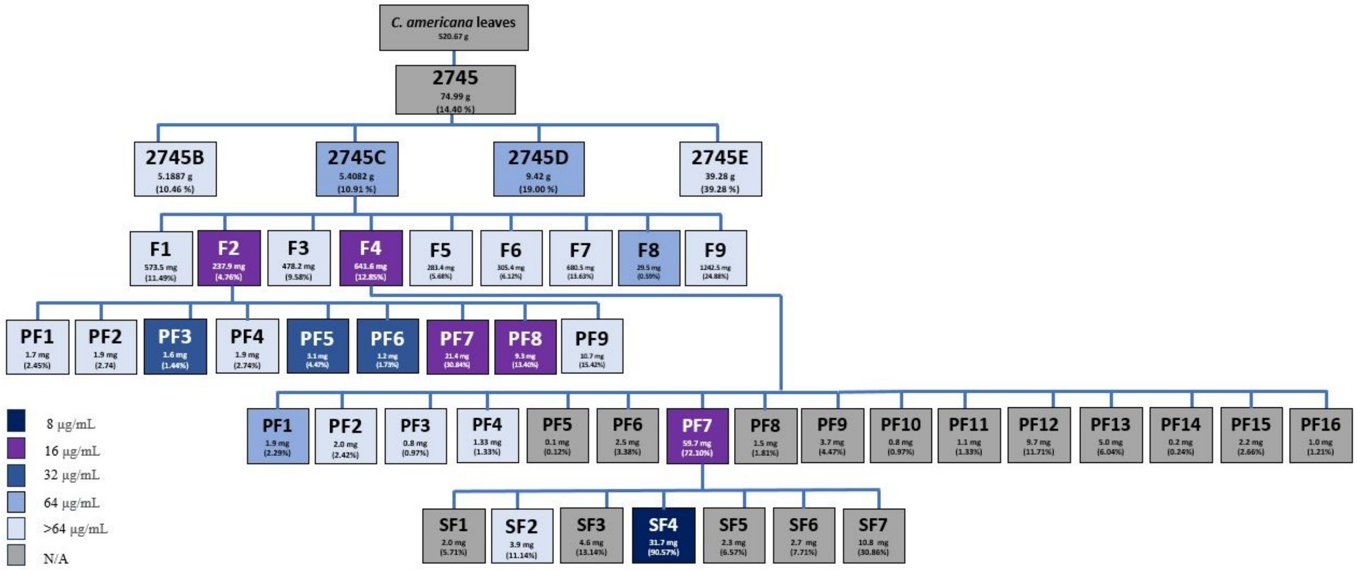

Figure S1: Fractionation tree of extract 2745. Schematic representation of bioassay-guided fractionation of *C. americana* leaf extracts against *C. acnes*. Color indicates the MIC_{90} values of *C. acnes*. Fractions without any color (gray) were not tested for growth-inhibitory activity. Total amount and percent yields are reported for each fraction, with the percent yield being calculated relative to its parent fraction.

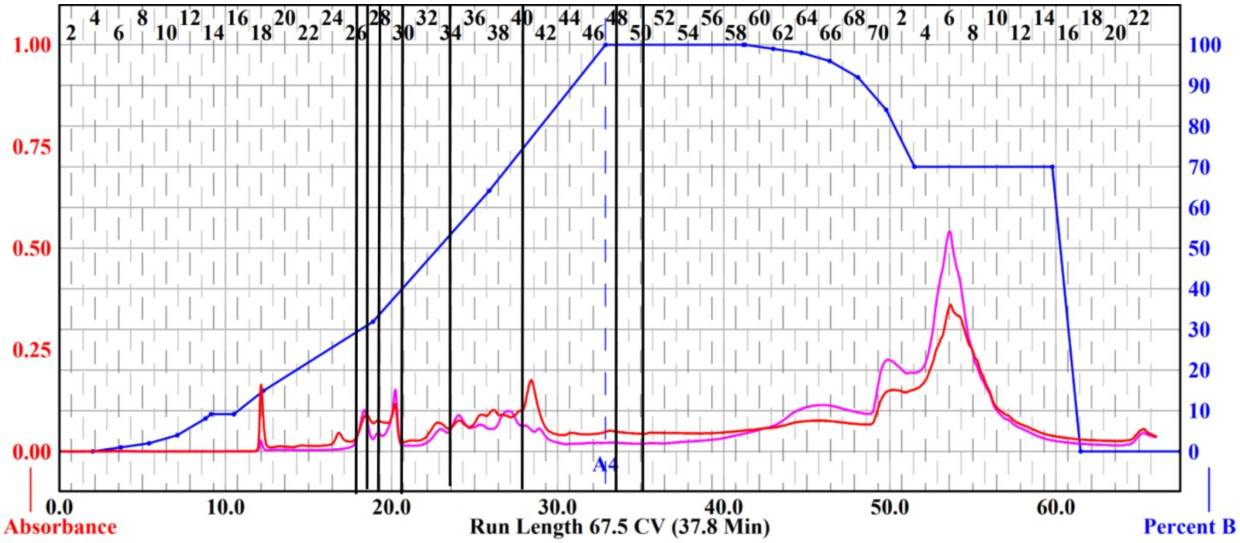

Figure S2: Flash chromatogram and separation scheme of 2745C. A 24 g Gold Silica column was used to separate fraction 2745C. Absorbance was measured at two wavelengths: 254 nm (red) and 320 nm (pink). A stepwise gradient mixture of three solvents was used, with the percentage of solvent B (ethyl acetate) represented in blue. The 93 initial fractions collected are represented by the dashed lines, while the solid lines represent how the final 9 fractions were separated.

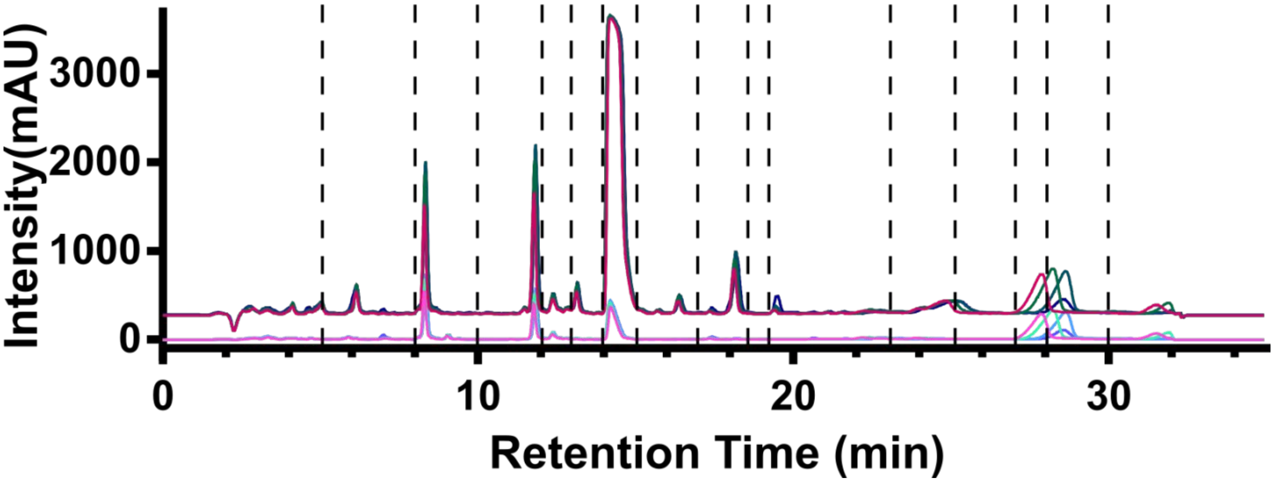

Figure S3: Reverse-phase HPLC chromatogram of 2745C-F4. HPLC was conducted using an Agilent Eclipse XDB-C18 (30 x 250 mm) column and a dual solvent system with 0.1% formic acid in water (v/v) and 0.1% formic acid in acetonitrile (v/v). Colored lines represent the absorbance readings at both 214 nm and 254 nm for five consecutive injections of 2745C-F4. Dashed lines represent how the fractions were separated for further bioassay-guided fractionation.

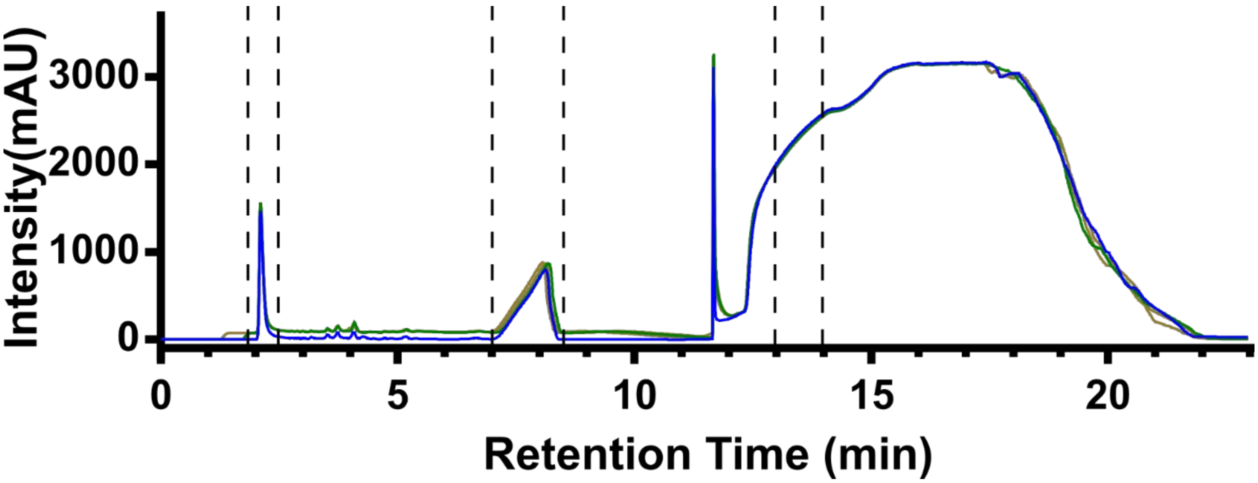

Figure S4: Normal-phase HPLC chromatogram of 2745C-F4-PF7. HPLC was conducted using a YMC-Pack SIL-06 10 x 250 mm column heated to 30.0°C with a dual solvent system of DCM:MeOH (97:3 v/v) and isopropyl alcohol (100%). Colored lines represent the absorbance readings at 254 nm for four consecutive injections of 2745C-F2. Dashed lines represent how the fractions were separated for further bioassay-guided fractionation.

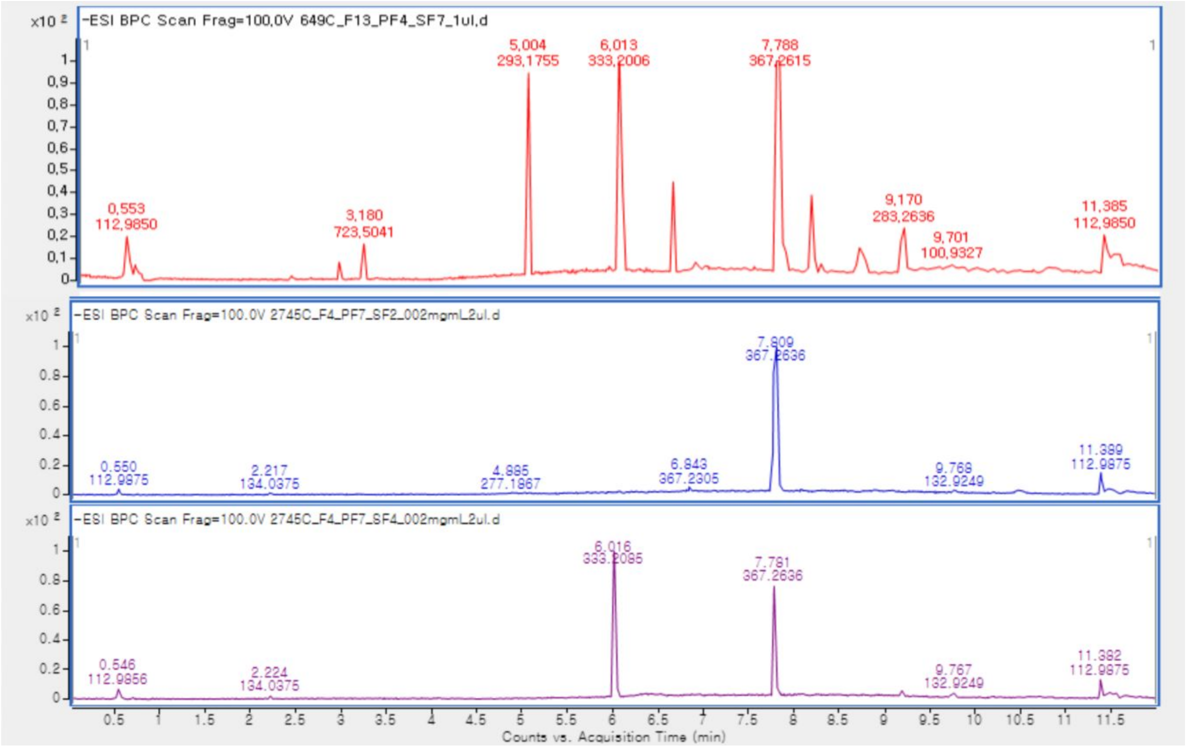

Figure S5: LC-MS chromatogram of *Callicarpa americana* leaf fractions. Mass spectra for three different *C. americana* leaf extract fractions are shown: 649C-F13-PF4-SF7 (red), 2745C-F4-PF7-SF2 (blue), and 2745C-F4-PF7-SF4 (purple). Y-axis represents relative intensity. All peaks are labeled with their respective retention times (above) and *m/z* value (below).

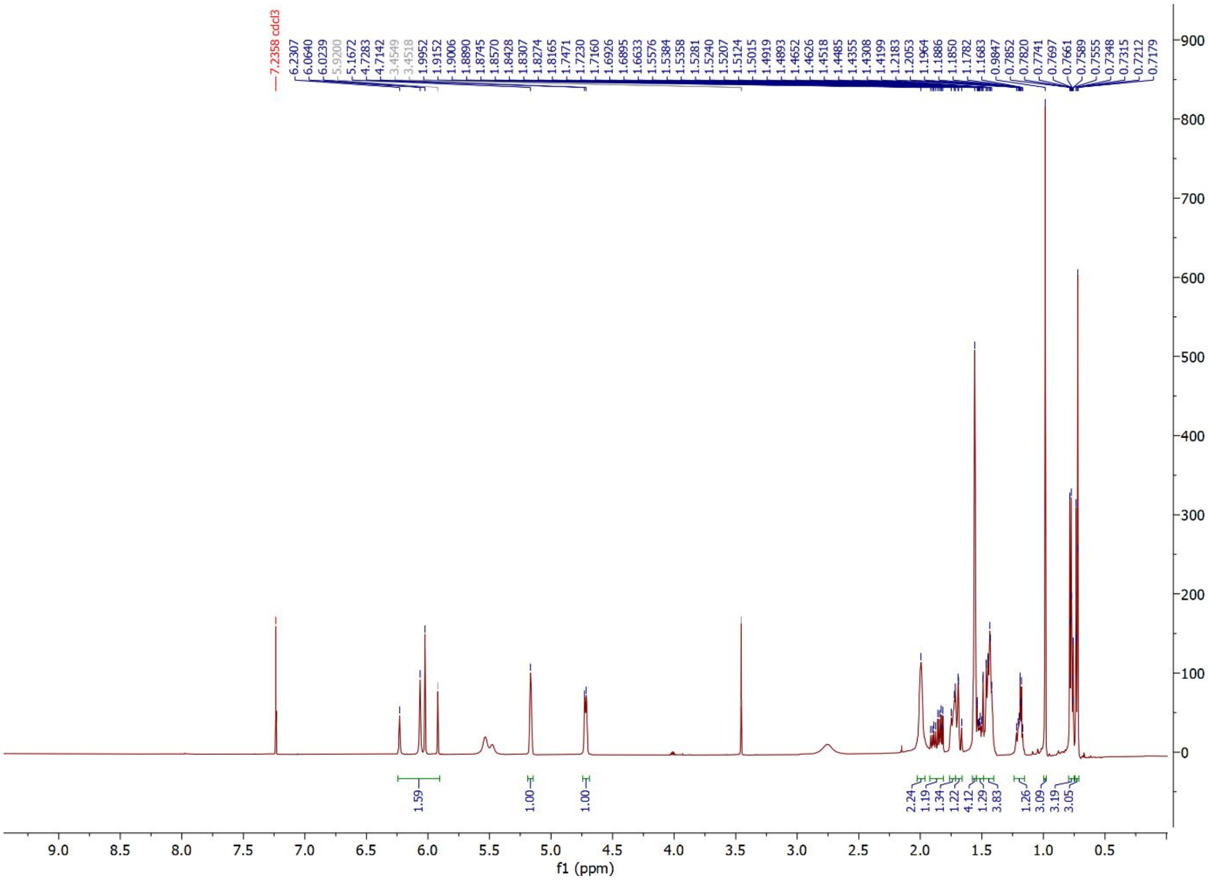

Figure S6. ^{1}H NMR Spectrum of 2745C-F4-PF7-SF4. 600 MHz, $CDCl_3$.

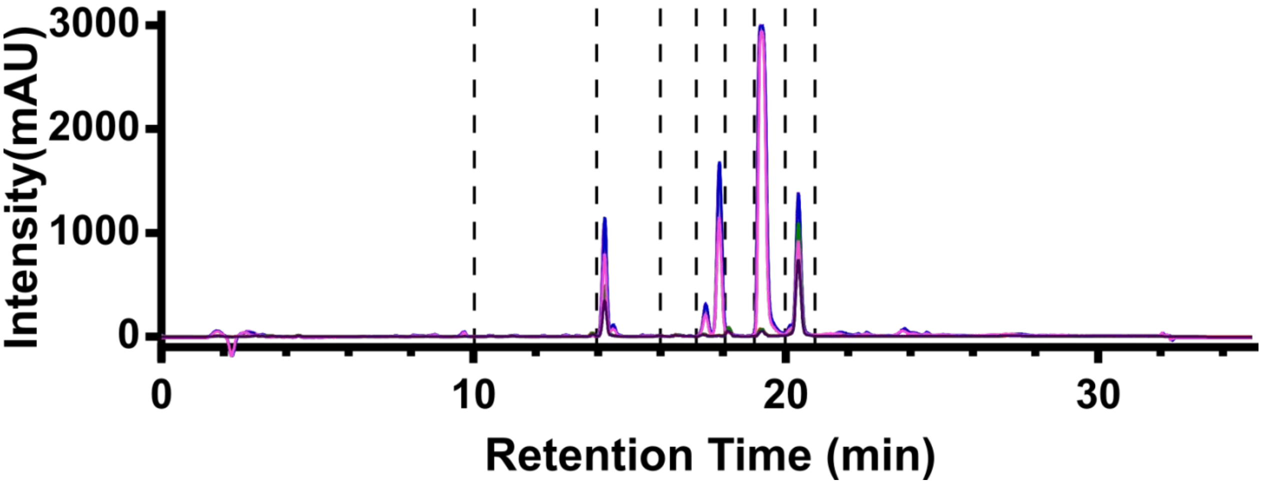

Figure S7: Reverse-phase HPLC chromatogram of 2745C-F2. HPLC was conducted using an Agilent Eclipse XDB-C18 (30 x 250 mm) column and a dual solvent system with 0.1% formic acid in water (v/v) and 0.1% formic acid in acetonitrile (v/v). Colored lines represent the absorbance readings at both 214 nm and 254 nm for three consecutive injections of 2745C-F2. Dashed lines represent how the fractions were separated for further bioassay-guided fractionation.
